# *Salmonella* Endocarditis: Rare Bacteremia Causing Mural Infective Endocarditis

**DOI:** 10.1155/crdi/3830316

**Published:** 2025-04-14

**Authors:** Seth Krueger, Michael Carcella, Caroline Dillon, Darrell McBride

**Affiliations:** ^1^Internal Medicine, Geisinger Medical Center, 100 North Academy Avenue, Danville 18722, Pennsylvania, USA; ^2^Pharmacology Infectious Disease, Geisinger Medical Center, 100 North Academy Avenue, Danville 18722, Pennsylvania, USA; ^3^Infectious Disease, Geisinger Medical Center, 100 North Academy Avenue, Danville 18722, Pennsylvania, USA

**Keywords:** cardiovascular implanted electronic device infection, endocarditis, gram-negative, mural infective endocarditis, *Salmonella*

## Abstract

Current guidelines do not recommend routine cardiac imaging in patients with gram-negative bacteremia, as gram-negative infective endocarditis is rare. Nongastrointestinal *Salmonella* infections causing endocarditis are even more uncommon, especially in the developed world. We present the case of a 60-year-old female with *Salmonella* bacteremia, ultimately found to have a right atrial mural endocarditis involving an implantable cardioverter–defibrillator and an indwelling venous catheter. The vegetation and indwelling devices were removed from the operating room due to the high concern of embolization if performed percutaneously, and she completed 6 weeks of antibiotic therapy. Both indwelling devices were later replaced once treatment was completed, and there was no evidence of recurrence at the 8-month follow-up. This case proves that in those with cardiac implantable electronic devices and other indwelling devices which enter the heart, who are found to have atypical bacteremia, may benefit from cardiac imaging as a part of their workup.

## 1. Introduction

Infective endocarditis (IE) is most commonly caused by gram-positive bacteria, such as *Staphylococcus, Streptococcus,* and *Enterococcus* species, and usually occurs in the immunocompromised, intravenous drug users, patients receiving hemodialysis, and those with structural cardiac abnormalities [1]. Rarely, IE can be caused by gram-negative bacteria, such as the *Salmonella* species. This gram-negative endocarditis is uncommon in the developed world; patients are often immunocompromised and the mortality rate is high [2]. Even more rare is mural IE (MIE), which is only described in case reports and a small number of retrospective reviews [3, 4]. In this report, we describe a patient who presented with septic shock due to *Salmonella* bacteremia and was ultimately diagnosed with *Salmonella* MIE, which involved her implantable cardioverter–defibrillator (ICD) and dialysis catheter. To our knowledge, this is the first case of its kind to be reported in the literature.

## 2. Case Presentation

**Figure 1:**
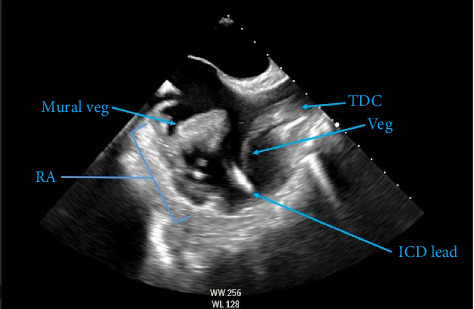
Transesophageal echocardiogram showing the right atrium (RA) with vegetations (veg) attached to the right atrial wall (mural veg) as well as intimately associated with a tunneled dialysis catheter (TDC) and implantable cardioverter–defibrillator (ICD) lead.

The patient was a 60-year-old Caucasian female with numerous medical comorbidities, most notable for end-stage renal disease on intermittent hemodialysis via tunneled dialysis catheter within the right internal jugular vein, and heart failure with reduced ejection fraction with in situ biventricular pacemaker/ICD. Other comorbidities included coronary artery disease, Type 2 diabetes mellitus, and chronic wounds of the right lower extremity. She presented to the emergency department with severe pain, swelling, and redness in the right lower extremity in the location of a chronic wound. She denied recent diarrheal illness or sick contacts. She was admitted to the hospital and treated with broad-spectrum antibiotics. Early in the admission, she developed signs of septic shock, prompting escalation to intensive care with vasopressor support. Preliminary culture data demonstrated growth of *Salmonella* species in all four blood culture bottles, with resistance to ampicillin and ceftriaxone, but susceptibility to cefepime, ciprofloxacin, and carbapenems. Computed tomographic (CT) imaging of the right lower extremity was normal and subsequent CT of the cervical, thoracic, and lumbar spine was also negative for osteomyelitis and discitis. An initial transthoracic echocardiogram (TTE) was performed and was unremarkable for intracardiac or valvular vegetations. Follow-up transesophageal echocardiography (TEE) demonstrated a 3.0 × 2.2 cm mass on the right lateral wall of the right atrium as well as multiple, scattered, mobile echo densities on the tip of the neighboring hemodialysis catheter (Figure 1). These findings were consistent with underlying endocarditis, likely due to *Salmonella*.

After receiving antibiotics, the patient was hemodynamically stable, and a multidisciplinary discussion was held between the patient and the primary medical team, electrophysiology, nephrology, and cardiovascular surgery. Options for management included definitive treatment with removal of the mass and contaminated hardware or long-term antibiotic suppression with frequent surveillance imaging. After a discussion of the risks and benefits, the patient underwent an unsuccessful attempt at transcatheter aspiration of the right atrial vegetation. She subsequently underwent an open cardiothoracic procedure for the removal of the mass. The ICD and dialysis catheter were also removed during surgery due to concern for increased risk of embolization if removed percutaneously. Intraoperatively, the large vegetation was found to involve the right atrial wall. Vegetations were also noted on the tip of the hemodialysis catheter, as well as on the implanted pacemaker leads. The tricuspid valve was not involved. No major complications occurred intraoperatively or in the postoperative course. Culture of the surgical specimens revealed *Salmonella* species, confirming the source of bacteremia.

Postoperatively, the patient was managed definitively with cefepime and completed 6 weeks of treatment. She was counseled on the risks of hardware reinfection versus the benefit of maintaining reliable hemodialysis access and ultimately chose for a replacement tunneled dialysis catheter in the right internal jugular vein. No recurrence was present on follow-up echocardiography at four and 8 months.

## 3. Discussion

*Salmonella* species are often associated with diarrhea [5]. However, in certain immunocompromised hosts, cardiac infection may occur [4, 6]. We have described a rare case of large MIE.

The incidence of isolated mural endocarditis is unknown but is regarded as less common than the valvular presentation and, in one study, represented only 0.07% of IE cases [4]. A retrospective analysis published in 2022 also found that vegetation in MIE is more likely to be large, defined as 10 mm or greater [3]. Patients with indwelling devices, such as central venous catheters or hemodialysis lines, have a higher incidence of mural involvement [4, 7, 8]. Gutierrez et al. hypothesized that the direct mechanical insult from a catheter tip or flow from the catheter jet predisposes local cardiac tissue to *Salmonella* colonization [4]. Our case is consistent with this hypothesis, as the catheter tip was intimately involved with the mural vegetation.

In addition to MIE, our patient also had a cardiovascular implanted electronic device infection (CIED). In the setting of CIED, extraction of the device is imperative and, when left in situ, there is a 7-fold increase in mortality within the first 30 days. With the proper extraction and antibiotic treatment, the risk of recurrence is 1% compared to 50%–100% when treated with antibiotics alone [9]. If the patient is considered too high risk to undergo removal, long-term palliative antibiotics can be considered, but this should be used as a last resort [10].

Since gram-negative endocarditis is rare, current guidelines do not recommend routine cardiac imaging in patients with gram-negative bacteremia, even in the setting of cardiac implantable devices [11]. We propose that cardiac imaging should be strongly considered when atypical bacteremia is found in patients who are receiving hemodialysis, are immunocompromised, or have indwelling devices. A thorough evaluation of endocarditis in these patients is imperative, as timely diagnosis is crucial in mitigating serious complications.

## Data Availability

Data sharing is not applicable to this article as no datasets were generated or analyzed during the current study.
